# Hyperexcitable Circuits in the Etiology of Psychosis in Alzheimer's Disease

**DOI:** 10.1002/alz70855_100356

**Published:** 2025-12-23

**Authors:** Matheus B Victor

**Affiliations:** ^1^ Icahn School of Medicine at Mount Sinai, New York, NY, USA

## Abstract

**Background:**

Approximately 40% of Alzheimer's disease (AD) patients develop psychotic symptoms (e.g. hallucinations and delusions), yet the neural processes that give rise to neuropsychiatric symptoms in dementia remain poorly understood.

**Method:**

To define the neurobiological correlates that distinguish AD patients with psychosis (AD+P) from AD patients that never exhibited psychotic symptoms (AD‐P), we performed single‐nucleus transcriptome and epigenome profiling from prefrontal cortex and hippocampus of 48 subjects segmented by psychiatric diagnosis.

**Result:**

Our snRNA‐seq profiling uncovered differentially expressed genes (DEGs) across multiple cell types, including a particular population of supragranular excitatory neurons in the AD+P cortex that exhibited transcriptional signatures of enhanced synaptic transmission, which we confirmed via in situ RNA hybridization in a separate cohort of AD+P postmortem brains. By leveraging our snRNA‐seq data with functional screens in stem‐cell derived brain organoids, we further define how genetic perturbation of AD+P DEGs modifies input‐output network connectivity in an *in vitro* model of cortico‐cortical communication. We identified that surviving excitatory neurons in upper cortical layers of AD+P brains re‐activate genetic programs associated with circuit assembly during development to remodel their neuronal network connectivity in the face of neurodegeneration.

**Conclusion:**

Our study suggests that this selective vulnerability of supragranular excitatory neurons may in part be driven by glial inflammatory programs triggered by differential layer‐specific neuropathological changes observed in AD+P. Unexpectedly, we found that these compensatory mechanisms may in fact further exacerbate pathology and network dysfunction. Our study provides novel insight into the pathophysiological role of hyperexcitable circuits in the etiology of neuropsychiatric symptoms of AD, and identifies new genetic targets in the search for new classes of antipsychotic treatments.

